# Molecular Crowding Defines a Common Origin for the Warburg Effect in Proliferating Cells and the Lactate Threshold in Muscle Physiology

**DOI:** 10.1371/journal.pone.0019538

**Published:** 2011-04-29

**Authors:** Alexei Vazquez, Zoltán N. Oltvai

**Affiliations:** 1 Department of Radiation Oncology, Robert Wood Johnson Medical School and The Cancer Institute of New Jersey, University of Medicine and Dentistry of New Jersey, New Brunswick, New Jersey, United States of America; 2 Department of Pathology, University of Pittsburgh, Pittsburgh, Pennsylvania, United States of America; University of Zaragoza, Spain

## Abstract

Aerobic glycolysis is a seemingly wasteful mode of ATP production that is seen both in rapidly proliferating mammalian cells and highly active contracting muscles, but whether there is a common origin for its presence in these widely different systems is unknown. To study this issue, here we develop a model of human central metabolism that incorporates a solvent capacity constraint of metabolic enzymes and mitochondria, accounting for their occupied volume densities, while assuming glucose and/or fatty acid utilization. The model demonstrates that activation of aerobic glycolysis is favored above a threshold metabolic rate in both rapidly proliferating cells and heavily contracting muscles, because it provides higher ATP yield per volume density than mitochondrial oxidative phosphorylation. In the case of muscle physiology, the model also predicts that before the lactate switch, fatty acid oxidation increases, reaches a maximum, and then decreases to zero with concomitant increase in glucose utilization, in agreement with the empirical evidence. These results are further corroborated by a larger scale model, including biosynthesis of major cell biomass components. The larger scale model also predicts that in proliferating cells the lactate switch is accompanied by activation of glutaminolysis, another distinctive feature of the Warburg effect. In conclusion, intracellular molecular crowding is a fundamental constraint for cell metabolism in both rapidly proliferating- and non-proliferating cells with high metabolic demand. Addition of this constraint to metabolic flux balance models can explain several observations of mammalian cell metabolism under steady state conditions.

## Introduction

The Warburg effect, i.e., glycolysis with lactic acid production even under normal oxygen saturation (aerobic glycolysis) concomitant with mitochondrial oxidative phosphorylation (OxPhos), is a metabolic phenotype displayed by most cancer cells [1]. The Warburg effect is also seen in dividing normal lymphocyte [2], [3], endothelial- [4], hair follicle [5] and fibroblast cells [6], [7], indicating that it is inherent to all rapidly proliferating mammalian cells [8]. Yet, the emergence of this mixed metabolic phenotype is seemingly counterintuitive, given that glycolysis produces only 2 moles of ATP per mole of glucose, far less than the 32 generated by OxPhos. Therefore, it has been argued that the Warburg effect represents a compromise between conflicting metabolic needs, in which beside the need for ATP the increased production of glycolytic intermediates is critical to satisfy the need of proliferating cells for biosynthetic precursor molecules [8], and that the high level of NADH produced during this enhanced glycolysis can be most efficiently converted back to NAD^+^ by the reduction of pyruvate to lactate [9].

However, anabolic processes may not represent the main factors underlying the Warburg effect because non-proliferating cells can also display similar metabolic phenotypes. In particular, the *lactate threshold* is a well known feature of muscle physiology, whereby muscle cells switch to partial anaerobic glucose catabolism when their contraction activity, and their corresponding ATP demand for converting chemical energy to mechanical work exceeds certain intensity [10], [11], even when oxygen abundance is not a limiting factor [12], [13], [14]. Also, in contrast to proliferating cells, anabolic processes are downregulated in heavily working muscles leading to decreased demand for biosynthetic precursors [15].

Taken together, the commonality between the Warburg- and lactate threshold effects is that the switch from aerobic- to mixed anaerobic-like metabolism takes place when the ATP production demand exceeds a threshold, although these demands satisfy different needs. Thus, in spite of their different routes of utilization, from the viewpoint of energy metabolism the main challenge is to understand the differential utilization of catabolic pathways as a function of the ATP demand. Here we address this fundamental issue by focusing on the interplay between the catabolism of glucose and fatty acids by a generic mammalian cell, by extending our previous model of glucose catabolism within the crowded intracellular milieu of proliferating cells [16]. The similarities and differences between the metabolisms of these cell types are further investigated using a larger scale model, accounting for the need of biosynthetic precursors in proliferating cells and amino acids catabolism.

## Results

### Reduced flux balance model of mammalian cell catabolism

**Figure 1:**
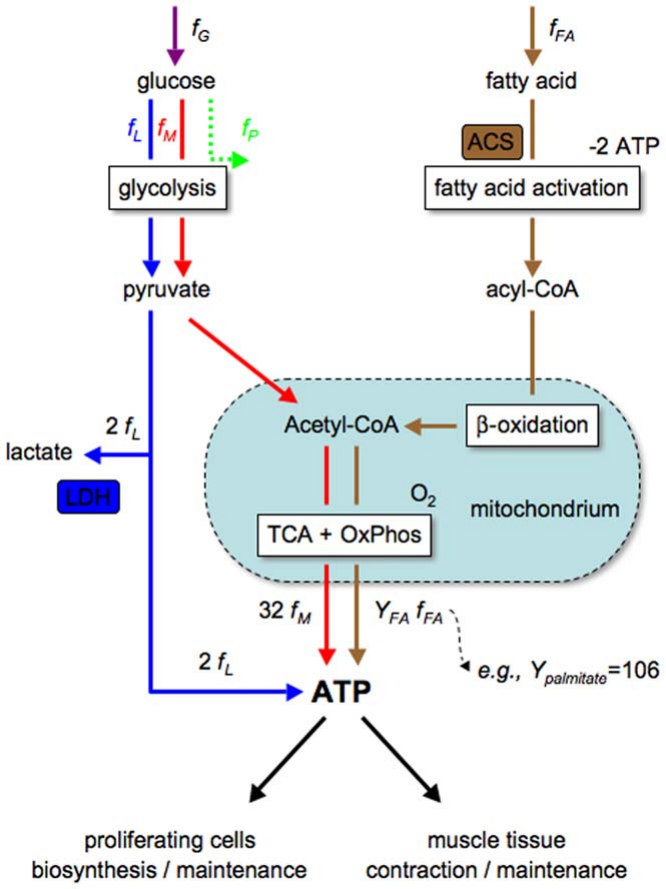
Reduced model of mammalian energy metabolism. Schematic representation of ATP generation pathways via anaerobic glucose catabolism (blue arrows, flux *f_L_*), aerobic glucose catabolism (red arrows, flux *f_M_*) and aerobic fatty acids catabolism (brown arrows, flux *f_FA_*). The green dotted arrow indicates an expanded variant of the model where we also take into account a third component of glucose fate, in which a fraction of the glucose flux is being directed toward the production of precursor metabolites (flux *f_P_*). LDH: lactate dehydrogenase, ACS: acyl-CoA synthetase, TCA: tricarboxylic acid cycle and OxPhos: oxidative phosphorylation.

Figure 1 depicts a schematic model of energy metabolism, including glucose- and fatty acid *catabolism* with pathway rates *f_G_* and *f_FA_*, respectively. The glucose utilization rate (*f_G_*) is partitioned into the anaerobic (*f_L_*) and aerobic (*f_M_*) glucose catabolism rates, where *f_G_* = *f_L_*+*f_M_*. Anaerobic glucose metabolism (*f_L_*) is decomposed into glycolysis, converting glucose into pyruvate, and then pyruvate reduction by lactate dehydrogenase (LDH) in the cytosol, resulting in the end product lactate that is then excreted to the extracellular milieu with a yield of *Y_L_* = 2 moles of ATP per mole of glucose. Aerobic glucose catabolism (*f_M_*) is decomposed into the glycolysis pathway producing pyruvate, the conversion of pyruvate to Acetyl-CoA, and the oxidation of Acetyl-CoA in the tricarboxylic acid (TCA) cycle coupled to OxPhos. Glycolysis yields *Y_M_* = 32 moles of ATP per mole of glucose [17], [18], two of which are produced in glycolysis and the other 30 from the TCA coupled to OxPhos in mitochondria. In the simplest case scenario, fatty acid (e.g., palmitate) catabolism is initiated by the acyl-CoA synthetase (ACS)-catalyzed activation of fatty acids to form acyl-CoA in the cytosol, β-oxidation of acyl-CoA to Acetyl-CoA in the mitochondria, and the oxidation of Acetyl-CoA in the TCA cycle coupled to OxPhos (Fig. 1). Fatty acid activation utilizes 2 ATP, while the catabolism of acyl-CoA yields *Y_FA_*+2 ATP, resulting in a yield of *Y_FA_* moles of ATP per mole of fatty acid (e.g., *Y_palmitate_* = 106). Summing up these contributions we obtain the rate of ATP production
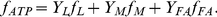
(1)


Our aim is to determine the optimal flux distribution (*f_L_*, *f_M_*, *f_FA_*) that provides a required *f_ATP_* demand given the cell's metabolic constraints. Of these, the first metabolic constraint is associated with the existence of a limited supply of nutrients, here denoted by *f_S_*, to fuel catabolic pathways [19]

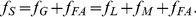
(2)This constraint takes into account that the utilization of the different nutrient pools requires common cellular resources, including membrane surface for the transporter molecule(s)-mediated entry of substrates into the cytosol, or cell volume for intracellular storage pools (e.g., of glycogen). Thus, preference for utilization of one type of nutrient (or substrate) will limit those resources for the utilization of other substrates.

The second constraint is the limited solvent capacity for the allocation of cytosolic enzymes and mitochondria [16]. In our case, this constraint applies to the cell volume occupied by glycolytic enzymes, LDH, ACS, and mitochondria. Enzymes associated with other pathways occupy a fraction of the intracellular volume as well. Nevertheless, this fraction simply restricts the amount of the cytoplasmic space available to glycolytic enzymes, LDH, ACS and mitochondria. More precisely, if *V_G_*, *V_LDH_*, *V_ACS_* and *V_M_* are the cell volume occupied by glycolytic enzymes, LDH, ACS and mitochondria, respectively, then *V_G_*+*V_LDH_*+*V_ACS_*+*V_M_*≤*V_ATP_*, where *V_ATP_* is the total cell volume available for the allocation of components of the ATP producing pathways. The occupied volumes *V_G_*, *V_LDH_*, *V_ACS_* and *V_M_* are proportional to the enzyme masses *M_G_*, *M_LDH_*, *M_ACS_* and the mitochondrium *M_M_*, with *V_G_* = *v_G_M_G_*, *V_LDH_* = *v_LDH_M_LDH_*, *V_ACS_* = *v_ACS_M_ACS_* and *V_M_* = *v_M_M_M_*, where *v_G_*, *v_LDH_*, *v_ACS_* and *v_M_* are the specific volumes of glycolytic enzymes, LDH, ACS and mitochondria, respectively. In turn, the glycolysis rate [*f_G_*], the lactate excretion rate [2*f_L_*], fatty acid activation rate [*f_FA_*] and the mitochondrial ATP production rate [(*Y_M_*−2)*f_M_*+(*Y_FA_*+2) *f_FA_*)] are proportional to the mass of glycolytic enzymes, LDH, ACS and mitochondria, respectively, with *f_G_* = *r_G_M_G_*/*V*, 2*f_LDH_* = *r_LDH_M_LDH_*/*V*, *f_ACS_* = *r_ACS_M_ACS_*/*V* and (*Y_M_*−2)*f_M_*+(*Y_FA_*+2)*f_FA_* = *r_M_M_M_*/*V*, where *r_G_* is the glycolysis rate per unit of glycolytic enzyme mass, *r_L_* is the rate of lactate production per mass of LDH, *r_ACS_* is the rate of fatty acid activation per mass of ACS, *r_M_* is the mitochondrial ATP production rate per unit of mitochondrial mass, and *V* is the cell volume. In these equations the product by the mass and the division by the cell volume takes into account that the rates *r* are commonly reported in the literature per unit of dry weight, while the pathway rates *f* are reported per unit of cell volume. Because of the interdependency of volume, mass and reaction rate, the volume constraint can be translated to the metabolic constraint
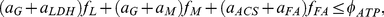
(3)where *φ_ATP_* = *V_ATP_*/*V* is the total volume fraction of the cell cytoplasm occupied by glycolytic enzymes, LDH, ACS and mitochondria, and the *crowding coefficients* [20] *a_G_* = *v_G_*/*r_G_*, *a_L_* = 2 *v_LDH_*/*r_LDH_*, *a_ACS_* = *v_ACS_*/*r_ACS_*, *a_M_* = (*Y_M_*−2) *v_M_*/*r_M_* and *a_FA_* = (*Y_FA_*+2) *v_M_*/*r_M_* quantify the occupied volume fractions per unit of glycolysis, lactate excretion, fatty acid activation and glucose and fatty acid associated mitochondrial respiration rate, respectively. Using empirical data reported in the literature we have estimated the crowding coefficients (see Methods). Regarding the glucose utilization pathways, we obtain *a_G_*≈0.0027 (min/mM), *a_LDH_*≈0.00023 (min/mM) and *a_M_* ≈0.09 (min/mM), indicating that mitochondria contribute about 5 to 50 times more to molecular crowding than glycolytic enzymes and LDH. Similarly, for the fatty acid oxidation pathway, *a_ACS_*≈0.00004–0.0002 (min/mM) and *a_FA_*≈0.3 (min/mM) (assuming that palmitate is the fatty acid substrate and *Y_FA_* = *Y_palpmitate_* = 106) and, therefore, mitochondria contribute at least 1,000 times more toward molecular crowding than ACS. Taken together these estimates indicate that the solvent capacity constraint (Equation (3)) can be approximated by *a_M_f_M_*+*a_FA_f_FA_*≤*φ_ATP_*. Note, we cannot exclude the possibility that under certain physiological conditions *a_G_*, *a_ACS_*, or *a_LDH_* may achieve values comparable to that of *a_M_*. Yet, for the sake of simplicity, we limit our analysis to the scenario supported by the current empirical evidence.

In our modeling we assume that *V_ATP_*, *r_G_*, *r_LDH_*, *r_ACS_* and *r_M_* are constant parameters that can be obtained from experimental estimates. Note though, that this is an approximation, as there may be regulatory mechanisms that under certain environmental or developmental conditions are capable of altering the amount of intracellular space allocated to ATP producing pathways and the activity of glycolytic enzymes, LDH, ACS and mitochondria. Also, note that our model can be easily modified to investigate scenarios where one or more pathways are not active due to the absence of expression of the pathway enzymes, by simply setting to zero the corresponding pathway rate. For example, the enzymes involved in fatty acid catabolism are generally not expressed or are expressed at low levels in cancer cells [21] and in this case *f_FA_*≡0.

Given the model (1)–(3), the relevant metabolic optimization problem can be formulated in two, mathematically equivalent ways. *ATP production maximization*: maximize the ATP production rate *f_ATP_* given the nutrient supply *f_S_*, subject to the solvent capacity constraint (3). In this case (1) is the optimization objective while (2) and (3) are constraints. *Supply cost minimization*: minimize the nutrient supply *f_S_* to satisfy a given ATP production demand *f_ATP_*, subject to the solvent capacity constraint. In this case (2) is the optimization objective while (1) and (3) are constraints. Furthermore, when solving these optimizations problems, we can use either *f_S_* or *f_ATP_* as a control parameter to investigate the existence of metabolic regimes with different flux distributions (*f_L_*, *f_M_*, *f_FA_*).

### Optimal solution for cell catabolism

In the following we discuss the model-predicted utilization of anaerobic glucose-, and aerobic glucose and fatty acid catabolism as a function of the ATP demand *f_ATP_*.

**Figure 2:**
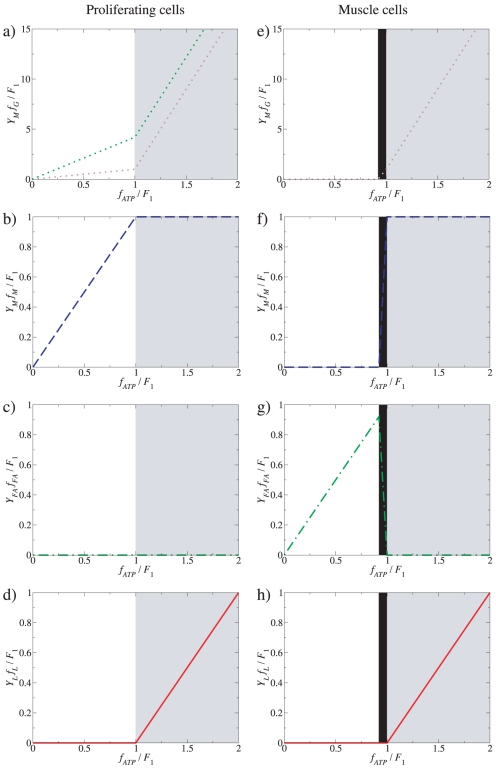
Catabolic regimes as a function of the ATP demand. Model-predicted fluxes as a function of the ATP demand assuming no limit on glucose and fatty acid uptake, in the absence (a–d) and presence (e–h) of fatty acid utilization (*f_FA_*). Total glucose- (a,e), mitochondrial glucose- (b,f) and mitochondrial fatty acid utilization (c,g) are shown, together with lactic acid production rates through aerobic glycolysis (d,h). The green dotted line in a) indicates the correction in the model predictions after considering that a component of the glucose flux is also being directed toward the production of precursor metabolites (Fig. 1, green dotted line, flux *f_P_*).

When *f_FA_*≡0 there are two different regimes (reported in [16], but as a function of glucose availability *f_G_*):
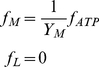
(4)for *f_ATP_*<*F*_1_, and for *f_ATP_*≥*F*_1_

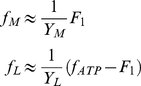
(5)where
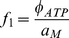
(6)

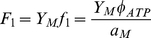
(7)and the ≈ symbol indicates that we have made use of the fact that *a_M_*>>*a_G_*, *a_LDH_*. This scenario is suitable to model the energy metabolism of rapidly-proliferating cells, including cancer cells, which are not using fatty acids as an energy source. The optimal solution is graphically illustrated in Figure 2a–d. From equation (4) it follows that at low glucose uptake rates (*f_G_*<*f*_1_) or low ATP demand (*f_ATP_*<*F*_1_), ATP is entirely produced by the aerobic pathway and there is no lactate production (nor excretion). This trend continues up to a threshold glucose uptake rate *f*_1_, or ATP demand *F*_1_, when mitochondria occupy the entire cell volume fraction (*V_ATP_*) available for ATP production pathways (Fig. 2b, blue dashed lines, white background, *glucose-limited* or *low ATP demand regime*). Beyond this threshold value the concentration of mitochondria, and therefore the rate of ATP production through OxPhos, cannot be increased further (Fig. 2b, blue dashed line, gray background). However, equation (5) predicts that an additional (and increased) glucose uptake may be diverted toward pathway(s) less efficient in terms of ATP yield per mole of glucose (Fig. 2a, purple dotted line, gray background, *solvent capacity-limited regime*). Indeed, the optimal solution predicts a metabolic switch, in which glucose uptake rates larger than *f*_1_ (defined in equation (6)), or ATP demand above *F*_1_ (defined in equation (7)), leads to a linearly increasing lactate production (Fig. 2d, red solid line, gray background, *solvent capacity-limited regime*), and therefore a component of the ATP production is now derived from the anaerobic pathway. This behavior was previously reported in Ref. [16], but now we emphasize that the switch may be equivalently driven by an increase of glucose uptake or ATP demand.

When *f_FA_* is unconstrained, and assuming that *Y_FA_* significantly exceeds *Y_M_* (e.g., *Y_palmitate_* = 106 whereas *Y_M_* = 32), there are three different regimes:
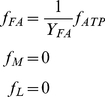
(8)for *f_ATP_*<*F*_0_,
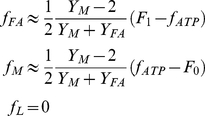
(9)for *F*_0_<*f_ATP_*<*F*_1_, and for *f_ATP_*≥*F*_1_

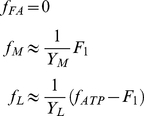
(10)where
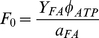
(11)and the ≈ symbol indicates that we have made use of the fact that *a_M_*>>*a_G_*, *a_LDH_* and *a_FA_*>>*a_ACS_*. This scenario is suitable for proliferating cells using both fatty acids and glucose as an energy source (e.g., prostate cells [21], [22]) and also for muscle metabolism. The optimal solution is graphically illustrated in Figure 2e–h. At low ATP demand (*f_ATP_*<*F*_0_), it is more efficient to produce ATP entirely from fatty acids, because this results in a higher ATP yield than either aerobic- or anaerobic glucose catabolism (Fig. 2g, white background). Fatty acid uptake increases until the mitochondrial capacity is saturated for ATP production from fatty acids, at *f_ATP_* = *F*_0_ (Equation 11). However, because fatty acid activation consumes 2 ATP molecules whereas aerobic glucose metabolism produces 2 ATP from the glycolysis step, mitochondrial respiration can satisfy a higher ATP demand by switching from utilization of fatty acids to glucose (Figs. 2f and g, dashed-grey background). This trend continues until the mitochondrial capacity is saturated for ATP production from glucose at *f_ATP_* = *F*_1_>*F*_0_ (Equation 7). For ATP demands beyond *F*_1_, we recover the same regimes as when setting *f_FA_*≡0, with the components *F*_1_ (Fig. 2f, gray background) and *f_ATP_*-*F*_1_ (Fig. 2h, gray background) from aerobic- and anaerobic glucose metabolism, respectively.

In addition to predict the shape of the plots in Figure 2, our model also predicts the threshold ATP demands where these transitions take place. Assuming that mitochondria occupy 7 to 38% of the cytoplasmic volume we obtain the threshold to lactate excretion *F*_1_ = 0.025–0.14 M/min (see Methods). In turn, the threshold for the transition from fatty acid to glucose can be shown to be proportional to *F*_1_ (from equations 7 and 11), *F*_0_ = [(*Y_FA_*(*Y_M_*−2))/((*Y_FA_*+2)*Y_M_*)]*F*_1_, where the coefficient of proportionality is determined by yield coefficients alone. For example, for palmitate *Y_palmitate_* = 106 and we obtain *F*_0_≈0.92*F*_1_, which was the value used to generate Figure 2d–f.

In proliferating cells, besides generating ATP the glycolysis pathway also plays a major role in providing precursor metabolites required for cell biomass production [8]. To investigate how the results of our analyses on ATP generation and cell catabolism are affected by accounting for the need of precursor metabolites, in a variant of our model we also introduce a third component of glucose fate, in which the glucose flux is also being directed toward the production of precursor metabolites, denoted by *f_P_* (Fig. 1). To take into consideration that proliferation will demand both precursor metabolites and energy in the form of ATP, let us denote by *D_P_*(*μ*) and *D_ATP_*(*μ*) the demand for precursor metabolites and ATP at a given proliferation rate *μ*. To satisfy these demands in a stoichiometrically balanced fashion we must thus have *f_P_* = *α*(*μ*)*f_ATP_*, where *α*(*μ*) = *D_P_*(*μ*)/*D_ATP_*(*μ*). This component of the glycolysis flux should thus be superimposed to that required to satisfy the ATP demand, as derived in the manuscript. This is illustrated in the Figure 2a, assuming that *α*(*μ*) = 0.1 and independent of the proliferation rate, just for the purpose of illustration. Importantly, this correction does not modify the results depicted in Figure 2b,d, plotting the component of glycolysis diverted towards oxidative phosphorylation and lactate excretion, respectively. Therefore, accounting for precursor metabolite needs does not qualitatively change the analytical results for the mode of ATP production under increasing metabolic demand.

### Larger scale flux balance model of mammalian cell metabolism

The reduced model described above is simple enough to gain a clear insight on the impact of the solvent capacity constraint on metabolism. On the other hand, these results need to be tested in the context of a larger scale model of cell metabolism. To this end, we expanded our analysis by considering a more detailed model of mammalian cell metabolism (see Methods). This model accounts for the catabolism of glucose, fatty acids, and amino acids (nutrients) and the synthesis of ATP and biomass components. For simplicity, fatty acids were represented by palmitate alone. We approximated the effect of the limited solvent capacity by imposing an upper bound on the mitochondrial respiration rate *f_R_*≤*f_R,max_*, where *f_R_* denotes the respiration rate and *f_R,max_* its maximum achievable value given the solvent capacity constraint. The latter can be estimated as *f_R,max_* = *F*_1_/*Y_ATP,R_* based on the calculations above, where *Y_ATP,R_* is the ATP production yield relative to the respiration rate, which is about 4.66 [23].

**Figure 3:**
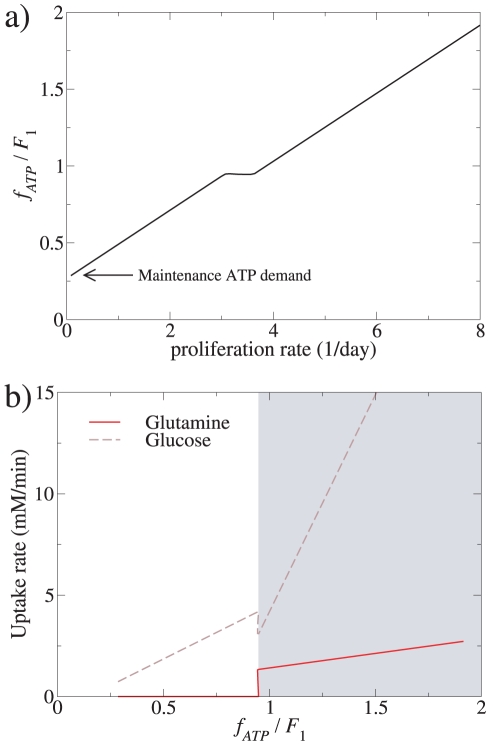
ATP demand in proliferating cells and nutrient uptake. a) Model-predicted ATP demand as a function of the proliferation rate (ln2/doubling time), calculated as the sum of the aerobic glycolysis and OxPhos ATP production rates. At zero proliferation rate the ATP demand corresponds to the maintenance needs as indicated by the arrow. Note the plateau at intermediate proliferation rates. b) Uptake rates of glutamine and glucose as a function of the ATP demand. It is evident that the plateau in a) and the transient drop in the glucose uptake rate are both associated with a sharp increase of the glutamine uptake rate and thus glutaminolysis.

**Figure 4:**
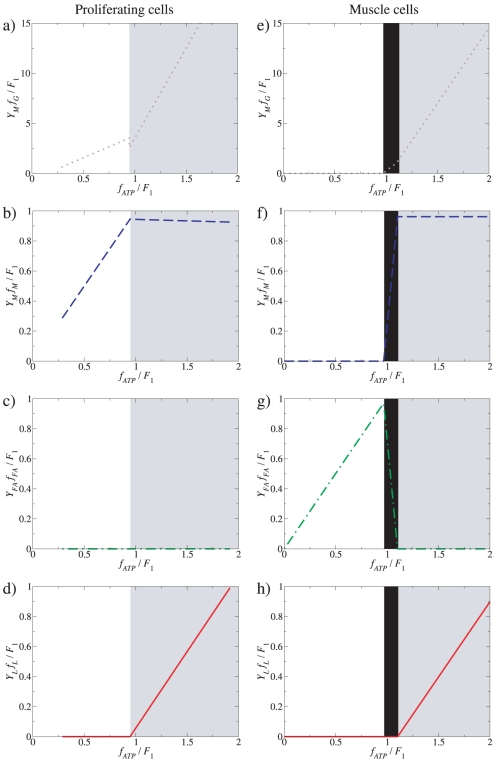
Catabolic regimes as a function of the ATP demand (full model). Model-predicted fluxes as a function of the ATP demand assuming no limit on glucose and fatty acid uptake, in the absence (a–d) and presence (e–h) of fatty acid utilization (*f_FA_*). Total glucose- (a,e), mitochondrial glucose- (b,f) and mitochondrial fatty acid utilization (c,g) are shown, together with lactic acid production rates through aerobic glycolysis (d,h).

To model the metabolism of proliferating cells we assumed that the major biomass cell components are produced at a rate proportional to the proliferation rate. Then we computed the optimal flux distribution minimizing the sum of nutrient uptake rates (glucose and amino acids) given a specified proliferation rate. We observed that the ATP demand, determined as ATP generated from aerobic glycolysis+mitochondrial OxPhos, increased with increasing the proliferation rate (Fig. 3a), although with an evident plateau at intermediate proliferation rates. This plateau was associated with a transient drop of the glucose uptake rate and a sudden increase of the glutamine uptake rate at ATP demands close to the threshold *F*_1_ (Fig. 3b), which is in agreement with the result of a recent study using a genome-scale metabolic model of mammalian cell metabolism [24]. Overall, the rate of glucose uptake *f_G_*, the rate of full glucose oxidation in the mitochondria *Y_M_f_M_*, and the lactate excretion rate *f_L_* manifest similar profiles (Fig. 4a–d) to those predicted by the reduced model (Fig. 2a–d). The metabolic switch takes place close to the threshold *F*_1_ predicted by the reduced model, although there is slight shift to the left.

To model the energy demands needed by muscle metabolism we used as before ATP generation as the metabolic objective and computed the optimal flux distribution minimizing the nutrients (glucose, fatty acids and amino acids) uptake given a specified ATP demand. The resulting glucose uptake, fatty acid uptake, full glucose oxidation and lactate excretion rates (Fig. 4e–h) manifested the same qualitative pictures as obtained with the reduced model (Fig. 2e–h). The metabolic switch takes place close to the threshold *F*_1_ predicted by the reduced model, although there is a slight shift to the right. We also noted that, in spite of the availability of amino acids, all energy was generated from fatty acids and glucose. Taking together these results validate the predictions from the reduced model in the context of a larger scale model of mammalian cell metabolism.

## Discussion

Empirical evidence indicates that the ATP generating capacity of OxPhos is saturated at physiological conditions with a high ATP demand. This is unequivocally deduced from the saturation of the oxygen consumption with increasing the muscle stimulation rate [25]. However, the precise mechanism behind the OxPhos saturation has been a topic of continuous debate. Limited oxygen availability could be an explanation and, indeed, oxygen intake saturates with increasing the running speed of human subjects [26]. However, the saturation in oxygen intake can just reflect a saturation of oxygen demand. Furthermore, experimental reports indicate that aerobic glycolysis of contracting muscles is independent of the oxygenation state [12], [13], [14]. A second hypothesis is the saturation of one or more enzymes in the TCA cycle and electron chain and, indeed, there is a linear correlation between maximum oxygen consumption and the maximum activity of at least one TCA cycle enzyme [27]. However, this correlation does not imply causality.

Here we provide evidence for an unavoidable physical constraint of biochemical systems as the origin for the activation of aerobic glycolysis: enzymes occupy a finite volume and, therefore, there is always an upper limit for the maximum rate for any metabolic pathway, which is roughly determined by the achievable maximum concentration of catalytic units, or more precisely by the solvent capacity constraint [16], [20], [28], [29]. Previous work [16], together with the results presented here indicates that the maximum ATP production rate is determined by the maximum mitochondrial concentration achieved at physiological conditions. Furthermore, ATP demands beyond that maximum must be satisfied by the activation of other pathways, e.g., aerobic glycolysis. Although aerobic glycolysis results in a lower ATP yield per mole of glucose it can achieve a higher ATP yield per volume occupied by the associated enzymes.

Importantly, we also show that our main results remain valid after considering a larger scale model of cell metabolism, including pathways for the generation of cell biomass components needed for cell proliferation. The results obtained with this model recapitulated the reduced model's predictions, reinforcing the notion that the ATP demand is the major driver of the Warburg effect. This observation contradicts the generally accepted notion that the need for precursor metabolites is the main driving force behind the Warburg effect in proliferating cells [8]. Moreover, while the increased need for precursor metabolites does certainly drive an increasing rate of glucose uptake (as shown analytically in Fig. 2a), it does not imply any switch-like behavior in the consumption of glucose, oxidative phosphorylation, and lactate excretion with increasing the proliferation rate. Also, if the need for precursor metabolites is really the limiting factor, then it would make no evolutionary sense to ‘waste’ most of the glycolysis flux towards the excretion of lactate.

On the other hand, the larger scale model, which accounts for the potential utilization of glucose and amino acids as well, uncovers a new observation. It predicts that the lactate excretion switch in proliferating cells coincides with activation of glutaminolysis. This prediction is in agreement with empirical observations indicating the activation of glutaminolysis as another major feature of the Warburg effect [30], [31], [32], [33] and with the result of a recent genome-scale metabolic model of mammalian cell metabolism [24]. Our model also predicts that concomitant with the activation of glutaminolysis, there is a small window of proliferation rates where the overall ATP production from aerobic glycolysis and mitochondrial OxPhos remains constant.

For ATP demands below the lactate threshold there are also differences associated with the use of fatty acids by contracting muscle cells. We predict that at low ATP demands fatty acids should be preferred over glucose, because they have a significantly higher ATP yield per mole of substrate. Thus the rate of fatty acid oxidation is predicted to increase linearly with increasing the ATP demand, until the maximum OxPhos capacity is reached. This is predicted to happen at an ATP demand below the lactate threshold, because fatty acid catabolism requires first the fatty acid activation that consumes two ATP, followed by their catabolism in the mitochondria. In contrast, glycolysis generates two ATP before being completely oxidized in the mitochondria. This difference results in a narrow window where OxPhos should shift from fatty acids to glucose as a substrate, until the lactate threshold is reached. These theoretical predictions are in perfect agreement with the experimental evidence that show an increase of fatty acid utilization with increasing muscle activity, reaching a maximum before declining completely to zero [34].

Our model has some limitations and cannot explain all aspects of proliferating normal or tumor cell- or contracting muscle cell metabolism. First, our analysis is based on a steady state approximation and, therefore, cannot account for events that occur on short time scales. For example, our model is useful to understand the optimal substrate utilization during steady-state muscle activity (e.g., running at constant speed) but cannot describe the transitions that occur during its initiation. Also, we predict that higher mitochondrial density will increase the maximum OxPhos capacity, but we cannot explain why the same tissue type (e.g., cardiac myocytes) from different species contains divergent mitochondrial densities. In addition, our does not explain why certain tissues (e.g., the brain) evolved not to utilize fatty acids at all.

In spite of these limitations, providing a common origin for the Warburg effect in cancer- and in all rapidly proliferating mammalian cells and the lactate threshold in muscle physiology significantly increases our understanding of human or mammalian cell metabolism and opens new research directions. Our modeling framework could also be used to anticipate how pharmacological interference with cancer metabolism would affect normal muscle cell physiology. In turn, we predict the activation of fatty acid utilization as mechanism for developing resistance to drugs targeting cancer glucose metabolism.

## Materials and Methods

### Parameters estimates

*a_G_*: The glycolysis rate per total mass of glycolytic enzyme is about *r_G_* = 0.29 mmol glucose uptake/min/g at 30°C [35], obtained as 23 µmol/min/mL lactate production rate (Table 4 in Ref. [35]), divided by 40 mg/mL of total glycolytic protein concentration (Table 2 in Ref. [35]), and divided by 2 to convert from lactate production to glucose uptake. The specific volume of glycolytic enzymes is estimated from the specific volume of globular proteins 0.79 mL/g [36], thus we use *v_G_* = 0.79 mL/g. Putting these two values together we obtain *a_G_* = *v_G_*/*r_G_* = 0.0027 min/mM. *a_L_*: The rate of lactate production per mass of lactate dehydrogenase is about *r_L_* = 7 mmol lactate/min/g at 30°C [35], obtained as 23 µmol/min/mL lactate production rate (Table 4 in Ref. [35]), divided by 3.2 mg/mL of lactate dehydrogenase concentration (Table 2 in Ref. [35]). The specific volume of lactate dehydrogenase is estimated from the specific volume of globular proteins 0.79 mL/g [36], thus we use *v_L_* = 0.79 mL/g. Putting these two values together we obtain *a_L_* = 2*v_L_*/*r_L_* = 0.00023 min/mM. *a_M_*: The mitochondrial rate of ATP generation per mitochondrial mass is in the range of 0.1–1.0 mmol ATP/min/g [37], [38], [39]. We use the maximum value *r_M_* = 1.0 mmol ATP/min/g. The mitochondrial specific volume has been reported to be 3.15 mL/g in mammalian liver [40] and 2.6 mL/g in muscle [41], respectively. We use the average between these two values *v_M_* = 2.9 mL/g. Putting these two values together we obtain *a_M_* = 32*v_M_*/*r_M_* = 0.09 min/mM and *a_FA_* = 106*v_M_*/*r_M_* = 0.3 min/mM (assuming *Y_FA_* = *Y_palmitate_* = 106). The fatty acid activation rate per unit of acyl-CoA synthase mass is different for different isoenzymes and substrates. For palmitate, *r_ACS_*≈17.5, 3.47 and 21.1 mmol/min/g, as have been reported for the rat acyl-CoA synthases ACS1, ASC3 and ASC4, respectively. The specific volume of acyl-CoA synthase is estimated from the specific volume of globular proteins 0.79 mL/g [36], thus we use *v_ACS_* = 0.79 mL/g. Using palmitate as a fatty acid substrate, we obtain *a_ACS_* = *v_ACS_*/*r_ACS_*≈0.00004–0.0002 (min/mM). *φ_ATP_*: The cell volume fraction occupied by cellular components involved in the generation of ATP was estimated from the cell volume fraction occupied by mitochondria. Literature reports for the latter range between 0.07 in some cell lines to 0.38 in muscle cells (Additional file 2 Table S1 in Ref. [16]). Thus we use *φ_ATP_* = 0.07–0.38. *f*_1_: Substituting the experimental estimates *a_M_* = 0.09 (min/mM), *a_ACS_*≈0.00004–0.0002 (min/mM), *Y_FA_* = *Y_palmitate_* = 106 and *φ_ATP_* = 0.07–0.38 into Equation 6, 7 and 11 we obtain *f*_1_≈0.78–4.2 mM/min, *F*_1_≈0.025–0.14 M/min and *F*_0_≈0.025–0.13 M/min, respectively.

### Large scale model

The large scale model of cell metabolism included the set of metabolic reactions listed in the Table S1, which were compiled from the BiGG database [42]. The biomass components were specified as their relative abundance in a generic mammalian cell [43]. In the case of proliferating cells their demand was specified as their abundance times the proliferation rate *μ*, the latter fixing the rate of the auxiliary reaction modeling biomass synthesis (Table S1, Biomass synthesis auxiliary reaction). Two other auxiliary reactions were added to account for the ATP needs for cell maintenance [43] and protein turn over [44] (Table S1, ATP maintenance and Protein turnover reactions). In the case of muscle cells an auxiliary reaction was added to simulate the ATP demand of muscle activity (Table S1, ATP synthesis reaction). The optimal flux distribution was computed solving the linear programming problem: minimize the sum of nutrient uptake rates (Table S1, Exchange fluxes, constrained), given a specified metabolic objective (proliferation rate or ATP demand) and a constraint on the maximum respiration rate. The respiration rate was determined as the sum of the cytochrome c oxidase, mitochondrial complex IV and complex III–IV, catalyzed reactions (Table S1, reactions 119 and 120). To apply the solvent capacity constraint we further assumed a mitochondrial volume fraction of *φ_ATP_* = 0.1, which following the derivation above results in *F*_1_ = 38 mM/min and *f_R,max_* = *F*_1_/*Y_ATP,R_* = 8 mM/min, where *Y_ATP,R_* = 4.66 is the ATP yield per respiration rate [23].

### Solvent capacity approximations

When making an estimate of the contribution of each pathway to the crowding of the cell cytoplasm we make use of the total sum of the enzyme masses that are necessary to maintain the pathway rate. To be fully precise, that would require the measurement of enzyme masses and pathway flux or, alternatively, the construction of a full metabolic model, to account for the metabolite concentration dependence of reaction rates. In the case of oxidative phosphorylation the rate of ATP production per mitochondrial mass is obtained from several experimental reports, which, because they are direct measurements, they already take into account all the metabolite regulations in the TCA cycle and electron transport chain. In the case of glycolysis, we have taken as a reference an experimental work reporting the glycolysis rate and the total sum of glycolytic enzymes masses. Thus, in the case of oxidative phosphorylation and glycolysis, we are not neglecting the role of metabolite concentrations since these are direct measurements. In the case of lactate excretion and β-oxidation of fatty acids we do assume that the reaction rates are proportional to the enzyme masses and neglect the regulation by enzyme concentrations. However, it is important to note that the latter approximation is only used to reach the conclusion that the contribution of lactate dehydrogenase and β-oxidation enzymes to molecular crowding is 10,000 smaller than the contribution of mitochondria. Therefore, although we have made an approximation, the profound difference between the estimated contributions to molecular crowding lead us to completely neglect the contribution of lactate dehydrogenase and β-oxidation enzymes to molecular crowding of the cell cytoplasm.

## Supporting Information

Table S1 List of reactions included in the larger scale model of mammalian cell metabolism.
(XLS)
